# Alignment of Borderline Personality Disorder and Complex Post-traumatic Stress Disorder With Complex Developmental Symptomatology

**DOI:** 10.1007/s40653-022-00445-6

**Published:** 2022-03-04

**Authors:** Jessica Lawless, Michael Tarren-Sweeney

**Affiliations:** School of Health Sciences, Canterbury University, Christchurch, New Zealand

**Keywords:** Out-of-home care, Developmental psychopathology, Complex disorders, Borderline personality disorder, Complex post-traumatic stress disorder, Classification of mental disorders, Child behavior checklist, Assessment checklist for adolescents

## Abstract

Cluster analysis of maltreatment-related mental health symptoms manifested by adolescents in foster care suggest the absence of an underlying taxonomic structure. To test this further, we investigated alignment between mental health symptom profiles derived through cluster analysis and nominal diagnosis of Borderline Personality Disorder (BPD) and Complex Post-traumatic Stress Disorder (C-PTSD), among a sample of 230 adolescents in long-term foster care. Nominal DSM-V BPD and ICD-11 C-PTSD caseness was estimated from Child Behaviour Checklist and Assessment Checklist for Adolescents score algorithms, and alignment of case assignment with previously-derived symptom profiles was examined. Nineteen BPD and three C-PTSD nominal cases were identified. Low C-PTSD prevalence reflected low concordance between PTSD and ‘disturbances in self organization’ (DSO) case assignment. The BPD and C-PTSD cases were aligned to more complex and severe symptom profiles. While the complex and severe presentations identified in the present study included core symptoms and clinical signs of BPD, they were also characterised by clinical-level inattention/over-activity and conduct problems. The present findings provide some support for the validity of the BPD construct for describing complex and severe psychopathology manifested by adolescents in foster care, and no support for the C-PTSD construct. However, the symptom profiles point to high variability in combinations of multiple symptom types that does not conform to traditional definitions of a ‘diagnosable’ mental disorder. Further research is needed to determine if complex post-maltreatment symptomatology can be validly conceptualised as one or more complex disorders.

## Introduction

Adolescents residing in long-term out-of-home care (OOHC) (foster, kinship and residential care) have generally experienced substantial, ongoing maltreatment from an early age, culminating in their entry into OOHC. Maltreated children and adolescents, including those placed into statutory care, frequently manifest complex mental health difficulties (DeJong, 2010; van der Kolk et al., 2005). These include combinations of: dysregulated affect and behaviour; inattention / over-activity; trauma-related anxiety, hypervigilance and re-experiencing trauma; dissociation and sensory disturbances; negative and unstable perceptions of oneself and others; attachment disorders and other attachment-related interpersonal difficulties; conduct problems, oppositional-defiance, low empathy and aggression; sexual behaviour problems; food maintenance behaviours (hoarding, storing, gorging); restricted, odd and stereotypic behaviours; depression; self-injury; and suicidal ideation and behaviour (Tarren-Sweeney, 2013b). Cumulative trauma exposure accounts for increasing symptom complexity in both childhood and adulthood (Cloitre et al., 2009). Clinicians therefore struggle to formulate such complex developmental symptomatology among these populations (Kocovska et al., 2012; Minnis, 2013). The accuracy and utility of clinical assessments of maltreated children and adolescents are thus compromised by clinicians’ inability to formulate complexity, such as resorting to co-morbid diagnosis (D'Andrea et al., 2012; Minnis, 2013); with incorrect formulation compromising the delivery of safe and effective treatments (Spinazzola et al., 2005; van Der Kolk, 2016). This also applies to assessment of complex symptomatology among *adults* who experienced chronic child maltreatment. Their complex presentations are typically formulated as co-morbid diagnosis of two or more of: Borderline Personality Disorder (BPD) (and other cluster B personality disorders); Post-traumatic Stress Disorder (PTSD); Attention-deficit / Hyperactivity Disorder (ADHD); Bipolar Disorder; Major Depressive Disorder; various dissociative disorders; and substance use disorders (Brand & Lanius, 2014; Creamer et al., 2001; Frías et al., 2016; Pagura et al., 2010; Weiner et al., 2019).

### Diagnostic Constructs that Capture Complex Symptomatology

#### Various Iterations of Complex PTSD

To what extent might complex post-maltreatment psychopathology be conceptualised within a typology of complex disorders? The most important work to date has focussed on differentiating the trauma symptomatology of adults who experienced childhood maltreatment, from that of adults exposed to discrete life threatening events (such as war or natural disasters or single instances of inter-personal violence). The PTSD construct arose from clinical studies of so-called ‘war neuroses’, and was formalised in the US following the Vietnam war (van der Kolk et al., 2005). The vast majority of cases who develop PTSD following exposure to war or discrete traumatic events in adulthood enjoy normative premorbid psychological development. Furthermore, research has shown that the trauma symptomatology of children, adolescents and adults exposed to severe and prolonged interpersonal trauma extends well beyond PTSD – reflecting more complex, ‘developmental’ adaptions to extreme stress, including neurobiological capacity to integrate cognitive, emotional and sensory information (van der Kolk, 2005). Indeed, it has been reported that a majority of traumatized children do not meet diagnostic criteria for PTSD (Cook et al., 2005).

To address this, a DSM-IV PTSD field trial was carried out in the early 1990s with the goal of better identifying disorders of extreme stress (Herman, 1992). Following a research review, the field trial workgroup proposed seven symptom categories not encompassed within the PTSD diagnosis, that are observed among people exposed to childhood trauma, women victims of domestic violence, and concentration camp survivors. These were: (a) dysregulation of affect and impulses; (b) alterations in attention or consciousness; (c) alterations in self-perception; (d) alterations in perception of the perpetrator; (e) alterations in relations with others; (f) alterations in systems of meaning; and (g) somatization (van der Kolk et al., 2005). The workgroup proposed a new diagnosis for inclusion in DSM-IV – Disorders of Extreme Stress Not Otherwise Specified (DESNOS) – supported by evidence from the field trials, and informally referred to as Complex PTSD (C-PTSD) (Herman, 1992). While the proposal was not adopted, DSM-IV listed the DESNOS symptoms as *associated and descriptive features* (American Psychiatric Association, 1994). Complex / developmental trauma constructs were further refined in the 2000s prior to DSM-V’s publication, and more recently in the run-up to ICD-11. Cook et al. (2005) refined the DESNOS categories and conceptualised seven domains of complex trauma (attachment; biology; affect regulation; dissociation; behavioural control; cognition; and self-concept). Van der Kolk and other members of the Complex Trauma taskforce of the U.S. National Child Traumatic Stress Network proposed a Developmental Trauma Disorder (DTD) for DSM-V, with four diagnostic criteria: (a) exposure to ‘developmental trauma’; (b) triggered pattern of repeated dysregulation in response to trauma cues (affective, somatic, behavioural, cognitive, relational, self-attribution); (c) persistently altered attributions and expectancies (with various specific examples); and (d) functional impairment (educational, familial, peer, legal, vocational) (van der Kolk, 2005).

While DESNOS and DTD were rejected for DSM-IV and DSM-V respectively, a further iteration of the construct, Complex PTSD (C-PTSD), was added to the ICD-11 (World Health Organization, 2020). ICD-11 C-PTSD has somewhat narrower diagnostic criteria than DESNOS and DTD. It is comprised of six symptom clusters grouped into two halves, namely ICD-11 PTSD diagnostic criteria (re-experiencing traumatic event; active avoidance of internal thoughts or external reminders; persistent perceptions of heightened current threat), and three ‘disturbances in self organization’ (DSO) criteria. The DSO criteria are severe and persistent: (1) problems in affect regulation; (2) beliefs about oneself as diminished, defeated or worthless, accompanied by feelings of shame, guilt or failure related to the traumatic event; and (3) difficulties in sustaining relationships and in feeling close to others (World Health Organization, 2020). C-PTSD diagnosis requires meeting all six PTSD and DSO criteria.

It is notable that the DESNOS relational difficulties category (‘alterations in relations with others’) does not specify some characteristic relational difficulties observed among maltreated children and adolescents, including secure base distortions such as role reversal and hyper-compliance, and various behaviours indicating non-attachment (Boris & Zeanah, 1999). Similarly, the ICD-11 C-PTSD criterion ‘persistent difficulties in sustaining relationships and in feeling close to others’ is rather narrowly defined, in that it excludes a range of characteristic relational difficulties seen among this population.

These omissions highlight the challenge in defining the amorphous developmental effects of childhood maltreatment. Chronically maltreated children are often exposed to multiple types of harmful developmental experiences that include physical and/or sexual abuse, as well as emotional abuse and emotional and physical neglect (Tarren-Sweeney, 2016). This goes some way to explaining the complexity of their psychopathology – the determinants of which involve complex interactions between trauma, attachment, genetic and social learning mechanisms.

#### BPD and Multiple Complex Developmental Disorder

BPD is an adult personality disorder characterised by a pervasive pattern of instability in affect regulation, impulse control, interpersonal relationships, and self-image (American Psychiatric Association, 2013). Clinical signs include emotional dysregulation, impulsive aggression and impulsivity in other areas, intensely unstable relationships, distorted cognition of relationships, mentalizing difficulties, self-injury, chronic feelings of emptiness or boredom, and chronic suicidal ideation and behaviour (Fonagy & Bateman, 2006; Newman & Stevenson, 2005; Trull et al., 2003).

Many of these behaviours and symptoms parallel those manifested by maltreated children and adolescents with complex developmental symptomatology. While BPD is not explicitly conceptualized as an attachment and/or trauma disorder, case control studies and surveys have established a strong link between BPD and childhood maltreatment and other adverse childhood experiences among both adult clinical (Helgeland & Torgersen, 2004) and adolescent samples (Belsky et al., 2012). One study estimated that 70–80% of adults with BPD have experienced trauma that is tied to their symptoms (Schmid et al., 2013). In particular, child sexual abuse is a common antecedent of BPD (Infurna et al., 2016; Venta et al., 2012). However, we still lack robust evidence of the developmental trajectory of borderline symptomatology from early childhood trauma through to adult BPD. For example, the long-term stability of emotional and behavioural dysregulation from early childhood through to early adulthood has not been adequately researched. There is also conflicting evidence of the stability of BPD symptoms in adolescence (Lewinsohn et al., 1997), with some studies indicating that adolescents’ symptoms are less continuous than those experienced by adults (Chanen et al., 2004; Levy et al., 1999). This may be partly accounted for by adolescents’ greater reactivity to present stressors and to the developmental demands of adolescence. This points therefore, to a risk of misidentifying adolescent crises as BPD, particularly among vulnerable youth with maltreatment histories and other co-existing mental health difficulties.

It is notable that several categories / domains specified in DESNOS, DTD and C-PTSD correspond to diagnostic features of BPD. The DESNOS workgroup did not set out to examine the PTSD or DESNOS constructs in relation to BPD (or other Axis I and Axis II disorders) (van der Kolk et al., 2005). However, ICD-11 C-PTSD was explicitly defined to be differentiated from BPD e.g. with respect to ‘fear of abandonment’ (not a feature of C-PTSD) and maladaptive self-image (BPD = shifting, unstable; C-PTSD = consistently negative) (Brewin et al., 2017; Cloitre et al., 2014). This may partially account for C-PTSD’s narrow symptom focus.

The ‘borderline’ construct has a somewhat messy and controversial history. It originated in psychoanalysis and has referred to various ideas, including psychopathology at the border between neurosis and psychosis, and early-onset childhood psychosis (Ad-Dab'bagh & Greenfield, 2001). Over time, ‘borderline’ and BPD labels have acquired a pejorative meaning, describing a class of emotionally dysregulated clients whose symptoms were thought to be untreatable, and who are difficult to engage with (McNab, McCutcheon, & Chanen, 2021). A BPD diagnosis conveys considerable stigma among mental health clinicians (Sansone & Sansone, 2013). While we might hope that the development of effective BPD treatments (such as Dialectical Behaviour Therapy and Mentalization-based Treatment) will improve clinicians’ attitudes towards people with BPD, applying a BPD label to maltreated adolescents, including those in OOHC, risks further stigmatising an already marginalised population (McNab et al., 2021). Consequently, if ‘adolescent BPD’ is ever confirmed to be a valid construct, then it warrants a better name – one that more accurately describes its developmental underpinnings. Several decades ago, scholars proposed a reconceptualization of child and adolescent ‘borderline’ symptomatology as a pervasive developmental disorder, which they termed ‘multiplex developmental disorder’ and ‘multiple complex developmental disorder’ (Ad-Dab'bagh & Greenfield, 2001; Cohen et al., 1986; Towbin et al., 1993). However, this idea has been subjected to very little empirical research.

### Empirical Investigation of Complex Developmental Symptomatology among Children and Adolescents

The above-mentioned diagnostic constructs consist of an historical adult diagnosis (BPD), and attempts to rectify the limitations of the PTSD diagnosis (DESNOS, DTD, C-PTSD). However, traditional conceptualizations of adult trauma syndromes may not be valid for children and adolescents. An alternative approach is to base our conceptualisation of complex post-maltreatment symptomatology on empirical data obtained using inductive research methods – rather than deductive modification of existing constructs. There have been remarkably few inductive investigations of complex developmental symptomatology among these populations. The validity of ICD-11 C-PTSD among children and adolescents has been investigated using finite mixture models (Latent Class Analysis; Latent Profile Analysis) that estimate probabilities of case membership of latent classes, notably PTSD and DSO (Ford, 2020; Haselgruber et al., 2020; Perkonigg et al., 2016; Sachser et al., 2017). These studies also had relatively narrow symptom coverage, limited to the PTSD and DSO constructs. They therefore employed a ‘top down’ confirmatory (i.e. deductive) approach. By contrast, ‘bottom up’ exploratory (i.e. inductive) analysis of complex symptomatology requires more comprehensive symptom coverage as well as *exploratory* statistical modelling. Cluster analysis is one such exploratory method, in which case clusters are identified using distance algorithms that seek to identify similar score profiles, without reference to pre-defined variables (Henry et al., 2005; Steele & Aylward, 2007). Cluster analyses of carer-report scores across broad symptom groupings (including general externalizing and internalizing symptoms, as well as symptoms more specific to maltreatment) have been carried out on Australian population samples of pre-adolescent children (*N* = 347) (Tarren-Sweeney, 2013b) and adolescents (*N* = 230) (Tarren-Sweeney, 2021) in family-based (foster and kinship) OOHC. Both child and adolescent cluster analyses yielded mental health profiles differentiated more by symptom severity and complexity, than by symptom specificity – suggesting that complex post-maltreatment symptomatology does not conform to a taxonomy of discrete disorders. Furthermore, 20% of the child sample (Tarren-Sweeney, 2013b) and 15% of the adolescent sample (Tarren-Sweeney, 2021) displayed complex maltreatment-related symptomatology that is not adequately conceptualized within DSM-5 or ICD-11 classifications.

### Alignment of Empirically Derived Symptom Profiles with BPD and C-PTSD

Whereas there are ongoing efforts to define and classify complex developmental symptomatology using conventional diagnostic classifications, empirical analyses of complex suggest it is not amenable to traditional taxonomic classification. This raises a question about the extent to which existing diagnostic constructs that encompass complex symptoms – notably C-PTSD and BPD – align with empirically derived symptom profiles. The present article reports secondary analyses of the aforementioned Australian adolescent mental health data published in this special issue (Tarren-Sweeney, 2021), designed to address the research question:

*“To what extent do DSM-V Borderline Personality Disorder and ICD-11 Complex Post-traumatic Stress Disorder align with complex symptomology manifested by adolescents in foster care?”*

## Method

### Study Design

The Children in Care study (CICS) was a prospective epidemiological study of the mental health and developmental risk exposure of children and young people in long-term foster and kinship care, in New South Wales (NSW), Australia. The CICS included a baseline cross-sectional survey of 4–11 year-olds conducted between November 1999 and January 2003 (*N* = 347); a follow-up survey of those participants conducted in 2009 (by which time they were adolescents); and a cross-sectional survey of additional adolescents carried out in 2011. The second and third of these surveys employed the same study design with a view to yielding a combined (*N* = 230) cross-sectional dataset obtained for a representative sample of adolescents residing in family-based OOHC (foster and kinship) in NSW. Data were collected from mail-out caregiver questionnaires, and from the state child welfare and OOHC database, except that child welfare records were not retrieved for newly-recruited adolescents. The caregiver questionnaire measured participants’ mental health, development, education and present status (e.g. type and makeup of present placement, recent life events).

The statutory agency that held legal guardianship of the young people approved the study, while participation in the adolescent survey also required informed consent of both the young person and their carer. Aside from providing informed consent, young people were not directly involved in the study.

The present analyses set out to investigate alignment between nominal BPD and C-PTSD case assignments and the empirically derived symptom profiles, with case assignment being estimated from mental health checklist score algorithms that correspond to diagnostic criteria.

### Mental Health Measures

Adolescent mental health was measured by two carer-report checklists, the Child Behaviour Checklist (CBCL, 2001 profile) (Achenbach & Rescorla, 2001) and the Assessment Checklist for Adolescents (ACA) (Tarren-Sweeney, 2013a). The CBCL measures common child mental health difficulties across eight empirically derived clinical subscales; two higher-order, broadband scales approximating spectrums of depressive/anxious symptoms (internalizing) and disruptive behavioural symptoms (externalizing); and a total problems score that provides a measure of global psychopathology. Additionally, the CBCL has six DSM-oriented scales derived through expert ratings of items’ conforming to DSM-IV-TR diagnostic criteria (Achenbach & Rescorla, 2001). CBCL scale score distributions are delineated into *normal*, *borderline clinical* and *clinical* ranges.

The ACA is a 105-item carer-report mental health rating scale, measuring behaviours, emotional states, traits, and manners of relating to others, as manifested by adolescents residing in OOHC (Tarren-Sweeney, 2013a). It was developed to measure a range of attachment- and trauma-related problems not adequately covered by standard survey instruments, including the CBCL, with most items being derived from the pre-adolescent Assessment Checklist for Children (Tarren-Sweeney, 2007). In addition to a total clinical score, the ACA has seven clinical scales that measure various attachment- and trauma-related symptomatology, derived through factor analysis of 73 core clinical items (a robust 7-factor model accounted for 51% of score variance). Four of the factors replicate ACC clinical scales (*non-reciprocal interpersonal behaviour*; *sexual behaviour problems*; *food maintenance behaviour*; and *suicide discourse*), and three are unique to the ACA (*social instability / behavioural dysregulation*; *emotional dysregulation / distorted social cognition*; and *dissociation / trauma symptoms*). The ACA also contains two low self-esteem scales (*low confidence*; *negative self-image*) that were constructed separately to the clinical scales. For each ACA scale, two cut-points were selected to identify young people with ‘possible’ and ‘probable’ clinical-level problems. Scores above the higher cut-points constitute *clinical* ranges that are highly predictive of psychiatric impairment (highly specific), while scores above the lower cut-point ranging up to and including the high cut-point constitute *elevated* ranges that retain high sensitivity for detecting psychiatric impairment. Initial data indicate that the ACA has good content, construct and criterion-related validity, as well as high internal reliability (Tarren-Sweeney, 2013a).

### Sample Recruitment

#### Follow-up Participants

Of 347 baseline survey participants, 231 remained in court-ordered foster or kinship care at follow-up. Of these, 66 were residing in placements that did not have a verifiable contact address, and whose carers could not be located by telephone. There were thus 165 young people that were eligible for inclusion in the follow-up survey, and could be reliably located. Of these, questionnaires were returned for 85 young people, representing a 51.5% response rate. However, these participants represented only 37% of baseline participants who remained in care.

#### Additional Adolescent Survey Participants

The sampling frame for recruiting additional adolescent survey participants was: young people aged 12 to 17 years residing in non-temporary court-ordered foster and kinship care in New South Wales, Australia; case supervision was provided by the statutory child welfare agency (i.e. not supervised by private agencies); were not part of the baseline survey sample; and whose placement address could be verified. Survey questionnaires were sent to the caregivers of 290 eligible participants residing at verified residential addresses with telephone contact, of which 145 were completed and returned (50% response rate). Additional adolescent participants did not differ to eligible non-participants in terms of age or gender distributions, geographical location, time in care, or age at entry into care.

### Sample Characteristics

The adolescent survey sample (*N* = 230) consisted of 85 follow-up participants, and 145 newly recruited participants. It included slightly more boys (54%, N = 125) than girls (46%, N = 105). Participants had a mean age of 15.3 years, ranging from 10.7 to 18.6 years. Ethnicity was not reliably recorded in the state database at the time data were collected, and hence it is not reported. Numbers of young people residing in foster and kinship care were 196 (85%) and 34 (15%) respectively. While the maltreatment histories of the newly-recruited participants were not retrieved, 79% and 75% of the follow-up participants had confirmed prior exposure to abuse (sexual, physical and/or emotional) and neglect respectively, while 55% had experienced both abuse and neglect. Aggregate sample mean CBCL and ACA scores have been reported previously (Tarren-Sweeney, 2018). Fifty-two percent of girls and 46% of boys had at least one CBCL syndrome or broadband scale score in the clinical range. Equivalent proportions of young people with any score in the borderline plus clinical ranges were 63% (girls) and 59% (boys).

### Symptom Profiles

**Fig. 1:**
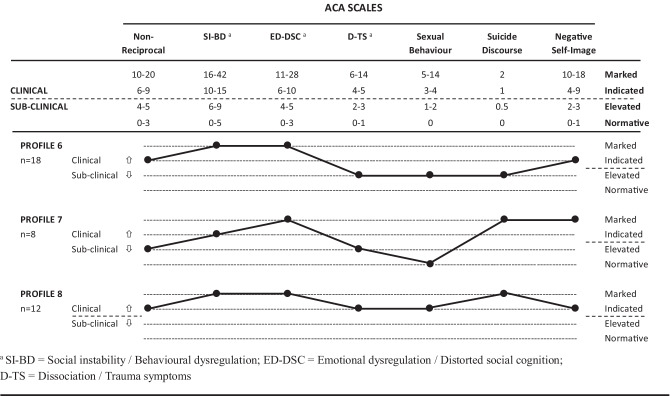
ACA symptom profiles #6, #7, and #8 ( Source: Tarren-Sweeney, 2021)

The companion article (Tarren-Sweeney, 2021) reports two sets of symptom profiles identified among the present sample through cluster analysis. The first cluster analysis identified symptom profiles across eight ACA scales for 113 ACA cases, defined by a clinical range score on one or more of the ACA scales, including the ACA total clinical score. Eight ACA symptom profiles (depicting variation in profile shape and mean scale score elevation) are listed in the companion article, while the three most severe and complex profiles (#6, #7, and #8) are listed in Fig. 1. The profiles place cluster mean scores for each of the ACA scales within the four ranges used on the ACA score profile sheets: sub-clinical scores are delineated between *normative range* and *elevated range* scores; while clinical-level scores are delineated between *clinically indicated range* (less severe) and *marked clinical range* (more severe) scores.

**Fig. 2:**
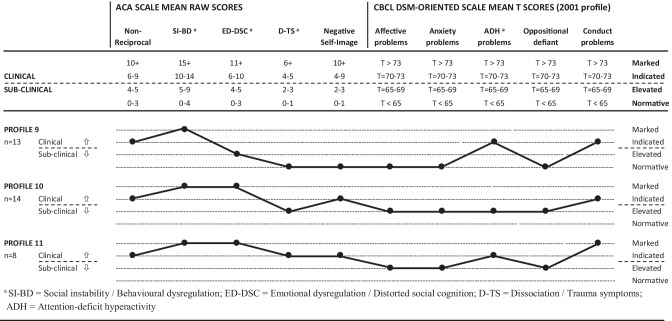
CBCL-DSM/ACA symptom profiles #9, #10, and #11 ( Source: Tarren-Sweeney, 2021)

The second cluster analysis sought to identify characteristic symptomatology across a broader range of symptoms, as measured across ten CBCL DSM-oriented and ACA scales, namely: five CBCL DSM-oriented scales (Affective problems, Anxiety problems, Attention-deficit / hyperactivity problems, Oppositional defiant problems, and Conduct problems); and five ACA scales (Nonreciprocal interpersonal behaviour; social instability / behavioural dysregulation; emotional dysregulation / distorted social cognition; dissociation / trauma symptoms; and negative self-image). Cases (*N* = 141) were defined as participants with a clinical range score on any CBCL broadband, CBCL DSM-oriented, or ACA scale. Symptom profiles for 11 CBCL-DSM/ACA clusters (depicting variation in profile shape and overall elevation of scores) are listed in the companion article, while the three most severe and complex profiles (#9, #10, #11) are listed in Fig. 2. For the CBCL-DSM scales, the four symptom severity ranges were defined by CBCL T-score ranges, namely *normative* (T-scores < 63, representing scores that are clearly normative); *elevated* (T-scores = 63–69, representing sub-clinical scores that are less clearly normative, with the upper end encompassing the CBCL borderline clinical range); *clinically indicated* (T-scores = 70–73, the moderate end of the clinical range); and *marked clinical* (T-scores > 73, the severe end of the clinical range).

### Defining Nominal BPD and C-PTSD Cases

Nominal BPD and C-PTSD cases were identified from a multi-step method. The first step was to identify and review all CBCL and ACA items that potentially match each diagnostic criterion. Where the two checklists have an equivalent item, the ACA item was selected. Most of the rejected items lacked sufficient specificity to the diagnostic criterion. The rejected items are listed in a ‘supplementary materials’ table, along with the reason for rejection. We could not identify any suitable items to measure C-PTSD criterion #2 (avoidance of thoughts and memories, etc.), which is one of the three PTSD diagnostic criteria. PTSD ‘caseness’ was therefore solely defined by criteria #1 and #3, which means that the number of PTSD cases was likely to have been over-estimated, and some participants who met criteria #1 and #3 are likely to have been incorrectly labelled as PTSD cases. Whereas BPD diagnostic criteria are adequately described in DSM-V, the C-PTSD diagnostic criteria listed on the ICD-11 website lack operational definitions, notably for the DSO criterion ‘difficulties in sustaining relationships and in feeling close to others’. We therefore applied the description listed by Cloitre et al. (2013, pp. 2–3) namely “individuals may consistently avoid, deride or have little interest in relationships and social engagement more generally. The person may occasionally experience close or intense relationships but will have difficulty maintaining emotional engagement”. The BPD diagnostic criteria are listed with the items selected to measure each criterion in Table 1, while the C-PTSD diagnostic criteria are listed with their selected items in Table 2.

**Table 1:** Items selected for BPD diagnostic criteria scales

**BPD diagnostic criteria**	**Criterion scale items**^a^
(1) Frantic efforts to avoid real or imagined abandonment	Extreme reaction to losing a friend, or being excluded by other young people ^b^ Fears you (or other adults) will reject him/her Is convinced that friends will reject him/her Possessive, can't share friends ^b^
(2) Pattern of unstable and intense interpersonal relationships characterized by alternating between extremes of idealization and devaluation	Changes friends quickly Extreme reaction to losing a friend, or being excluded by other young people ^b^ Possessive, can't share friends ^b^ Turns friends against each other
(3) Identity disturbance: markedly and persistently unstable self-image or sense of self	Seems like a completely different person (dramatic change in personality) (*pilot item*) Thinks he/she is someone or something else (*pilot item*)
(4) Impulsivity in at least two areas that are potentially self-damaging	Constantly seeking excitement or ‘thrills’ Impulsive (acts rashly, without thinking) Risks physical safety, fearless
(5) Recurrent suicidal behaviour, gestures, or threats, or self-mutilating behaviour	Attempts suicide Causes injury to him/herself Describes how he/she would kill him/herself Hits head, head-banging Intentionally harms him/herself with knife or sharp implement Threatens to injure him/herself Threatens to kill him/herself Talks about killing self (*CBCL*)
(6) Affective instability due to a marked reactivity of mood (e.g., intense episodic dysphoria, irritability, or anxiety usually lasting a few hours and only rarely more than a few days)	Extreme reaction to minor event (or for no obvious reason) Extreme reaction to losing a friend, or being excluded by other young people ^b^ Intense reaction to criticism Sudden or extreme mood changes
(7) Chronic feelings of emptiness	Says he/she feels ‘empty’ or without emotions
(8) Inappropriate, intense anger or difficulty controlling anger (e.g., frequent displays of temper, constant anger, recurrent physical fights)	Shows intense and inappropriate anger Uncontrollable rage Temper tantrums or hot temper (*CBCL*)
(9) Transient, stress-related paranoid ideation or severe dissociative symptoms	*A: Paranoid ideation:* Feels others are out to get him/her (*CBCL*) Says friends are against him/her *B: Severe dissociative symptoms* Appears dazed, ‘spaced out’ (like in a trance) Can’t tell if an experience is real or a dream Feels like things, people or events aren’t real Has periods of amnesia (e.g. has no memory of what has happened in the last hour)

^**a**^ACA items except where indicated in brackets. ‘CBCL’ indicates Child Behavior Checklist item; ‘Pilot item’ refers to item included in initial development study that was not retained in final version of the ACA

^b^Item included in more than one criterion scale

**Table 2:** Items selected for C-PTSD diagnostic criteria scales

**C-PTSD diagnostic criteria**	**Criterion scale items**^a^
**A.PTSD criteria**	
(1) Re-experiencing traumatic event or events in the present in the form of vivid intrusive memories, flashbacks or nightmares. These are typically accompanied by strong or overwhelming emotions, particularly fear or horror, and strong physical sensations	Can’t get scary thoughts or images out of his/her head Distressed or troubled by traumatic memories Nightmares about specific events or people
(2) Avoidance of thoughts and memories of the event or events, or avoidance of activities, situations, or people reminiscent of the event or events	*Nil items*
(3) Persistent perceptions of heightened current threat, for example as indicated by hypervigilance or an enhanced startle reaction to stimuli such as unexpected noises	Fears he/she might be molested (*pilot item*) Is fearful of being harmed Startles easily (‘jumpy’) Wary or vigilant (over-alert to danger)
**B.Disturbances in self organisation criteria**	
(4) Severe and pervasive problems in affect regulation	*A. Over-reactive* Causes injury to him/herself Extreme emotional reaction to minor event (or for no obvious reason) Intentionally harms him/herself with knife/implement Shows intense and inappropriate anger Sudden or extreme mood changes Temper tantrums or hot temper (*CBCL*) Uncontrollable rage *B. Under-reactive / dissociation* Appears dazed, ‘spaced out’ (like in a trance) Can’t tell if an experience is real or a dream Does not show pain if physically hurt Feels like things, people or events aren’t real Says he/she feels ‘empty’ or without emotions
(5) Persistent beliefs about oneself as diminished, defeated or worthless, accompanied by deep and pervasive feelings of shame, guilt or failure related to the traumatic event	Complains of not being likeable Feels ashamed Feels too guilty (*CBCL*) Feels worthless or inferior Says he/she is ‘bad’ or ‘no good’ Thinks other young people are better than him/her
(6) Persistent difficulties in sustaining relationships and in feeling close to others	Changes friends quickly Distrusts friends Does not show affection Seems alone in the world (not connected to people or places Won’t communicate with other young people (*pilot item*)

^**a**^ACA items except where indicated in brackets. ‘CBCL’ indicates Child Behavior Checklist item; ‘Pilot item’ refers to item included in initial development study that was not retained in final version of the ACA

The second step was to generate continuous scores for each criterion by adding the item scores. Each criterion thus had one continuous measure (i.e. a criterion scale), with the exceptions of BPD criterion #9 and C-PTSD criterion #4, which each required two criterion scales. Given that BPD criterion #9 is met if ‘transient, stress-related paranoid ideation *or* severe dissociative symptoms’ occur, we created separate scales to measure paranoid ideation and severe dissociative symptoms. Similarly, C-PTSD criterion #4 (Severe and pervasive problems in affect regulation) specifies two types of affect dysregulation (‘under-active / dissociation’ and ‘over-active’), and thus separate scales were created for both types.

The third step was to select a cut-point for each criterion scale score that defined if the diagnostic criterion is met or not. Cut-points were selected conservatively, with a view to identifying *probable* rather than *possible* cases. Cut-point selection was guided by examining the number of items in the respective criterion scale, the scale score distributions, and clinical judgement about item quality (e.g. item specificity for the criterion). For BPD criterion #9 and C-PTSD criterion #4, the criterion was met if either or both of the criterion sub-types were met.

Finally, nominal BPD and C-PTSD cases were defined by applying the relevant diagnostic rules. DSM-V BPD diagnosis is defined as meeting five or more BPD diagnostic criteria, whereas C-PTSD diagnosis requires that all five of the measured criteria are met. In addition to defining C-PTSD cases, we also separately defined PTSD caseness (criteria #1 and #3) and ‘disturbances in self organisation’ (DSO) caseness (criteria #4, #5 and #6). Statistical analyses were performed with STATA – version 16 (StataCorp, 2019).

## Results

BPD and C-PTSD criterion score means, diagnostic cut-points, and numbers and rates of participants meeting criteria thresholds, are listed in Table 3. Rates of participants meeting criteria thresholds ranged from 2.2% (BPD #7 ‘chronic feelings of emptiness’) to 30% (C-PTSD #4 ‘affect dysregulation’). The proportions of participants who met zero through to nine BPD diagnostic criteria, and zero through to five C-PTSD criteria are listed in Table 4. For both disorders, more than half of the participants met zero diagnostic criteria. Nineteen nominal BPD cases (those who met five or more diagnostic criteria) and three nominal C-PTSD cases (those who met all five diagnostic criteria) were identified.

**Table 3:** BPD and C-PTSD criterion score means, diagnostic cut-points and assignment rates (N = 230)

**Diagnostic criteria**	**N. criterion** **scale items** **(max score range **^**a**^**)**	**Mean criterion score**	**Diagnostic** **cut-point**	**Participants meeting criterion threshold**	
				**N**	**Rate**
***BPD criteria***					
(1) Attempts to avoid real or imagined abandonment	4 (0–8)	0.84	3 +	26	11.3%
(2) Unstable and intense interpersonal relationships	4 (0–8)	0.82	3 +	25	10.9%
(3) Identity disturbance	2 (0–4)	0.19	2 +	13	5.7%
(4) Self-damaging impulsivity	3 (0–6)	1.46	3 +	52	22.6%
(5) Recurrent suicidal / self-mutilating	8 (0–16)	0.52	2 +	23	10.0%
(6) Affective instability due to marked mood reactivity	4 (0–8)	1.48	4 +	38	16.5%
(7) Chronic feelings of emptiness	1 (0–2)	0.03	1 +	5	2.2%
(8) Inappropriate, intense anger / difficulty controlling	3 (0–6)	1.57	4 +	39	17.0%
(9) Transient, stress-related paranoia or dissociation			9a *OR* 9b	49	21.3%
*(9a) Paranoid ideation*	2 (0–4)	0.57	2 +	39	17.0%
*(9b) Dissociative symptoms*	4 (0–8)	0.62	3 +	18	7.8%
***C-PTSD criteria***					
**A. PTSD**					
(1) Re-experiencing traumatic event(s)	3 (0–6)	0.50	2 +	28	12.2%
(2) Avoidance of traumatic event(s)	No scale				
(3) Persistent perceptions of heightened current threat	4 (0–8)	0.55	2 +	37	16.1%
**B. Disturbances in self organisation**					
(4) Severe and pervasive problems in affect regulation			4a *OR* 4b	69	30.0%
*(4a) Over-reactive affect*	7 (0–14)	2.43	4 +	61	21.7%
*(4b) Under-reactive affect / dissociation*	5 (0–10)	0.67	3 +	18	7.8%
(5) Negative self-evaluation	6 (0–12)	1.04	4 +	20	8.7%
(6) Difficulties sustaining relationships	5 (0–10)	1.46	4 +	25	8.9%

^a^’Max score range’ refers to the maximum possible scale score range, given that items are scored 0,1,or 2

**Table 4:** Number of BPD and C-PTSD diagnostic criteria met (N = 230)

N. BPD criteria	N	%		N. C-PTSD criteria	N	%
0	129	56.1%		0	123	53.5%
1	39	18.0%		1	40	17.4%
2	23	10.0%		2	36	15.7%
3	8	3.5%		3	19	8.3%
4	12	5.2%		4	9	3.9%
*Diagnostic threshold*				*Diagnostic threshold*		
5	10	4.4%		5	3	1.3%
6	2	0.9%				
7	5	2.2%				
8	2	0.9%				
9	0	0.0%				

Table 5 reveals very little overlap between PTSD and DSO caseness. Among 22 participants who were PTSD and/or DSO cases, there were only three C-PTSD cases. BPD caseness is tabulated against PTSD, DSO and C-PTSD caseness in Table 6. This reveals moderately high overlap between DSO and BPD case assignment (62% of DSO cases were also BPD cases), and somewhat lower overlap between PTSD and BPD case assignment (25% of PTSD cases were also BPD cases). Two of the three C-PTSD cases were also BPD cases.

**Table 5:** Number of participants meeting PTSD and DSO diagnostic thresholds (N = 230)

		**PTSD threshold**		
**DSO threshold**		*Not met*	*Met*	*Total*
	*Not met*	208	9	217
	*Met*	10	3	13
	*Total*	218	12	230

**Table 6:** Overlap between BPD and C-PTSD case assignment

		**BPD case assignment**	
		*Non-case*	*Case*
	*Meets neither* *dx threshold (N* = *208)*	198	10
**C-PTSD** **case assignment**	*Meets PTSD* *dx threshold (N* = *12)*	9	3
	*Meets DSO* *dx threshold (N* = *13)*	5	8
	*C-PTSD cases (N* = *3)*	1	2

There are too few C-PTSD cases to report summary data. BPD cases and non-cases had the same mean age of 15.3 years. BPD prevalence among boys and girls was 6.4% (8/125) and 10.5% (11/105) respectively (Chi^2^ = 1.3, p = 0.26). BPD cases had exceptionally high mean mental health difficulties, both in absolute terms and in comparison with the other clinical cases in the present study. Among cases selected for the ACA-CBCL cluster analysis (*N* = 141), mean ACA total clinical scores for BPD cases versus other cases were 68.8 and 26.3 respectively (p < 0.0001), while mean CBCL total problems scores were 102.2 versus 54.0 respectively (p < 0.0001). Based on caregivers’ reports, twice as many BPD cases were prescribed psychotropic medication than were other clinical cases (59% and 30% respectively, *p* = 0.02).

Alignment of BPD and C-PTSD nominal diagnoses with the empirically derived ACA and DSM-CBCL/ACA symptom profiles are shown in Tables 7 and 8 respectively.

**Table 7:** Alignment of BPD and C-PTSD diagnoses with ACA symptom profiles

Profile	Total ACA cases (*N* = 113)	BPD cases (*N* = 19)	C-PTSD cases (*N* = 3)
#1	17	0	0
#2	21	0	0
#3	13	0	0
#4	15	1	0
#5	9	0	0
#6	18	4	1
#7	8	2	0
#8	12	12	2

**Table 8:** Alignment of BPD and C-PTSD diagnoses with CBCL-DSM/ACA symptom profiles

Profile	Total CBCL-DSM / ACA cases (*N* = 141)	BPD cases (*N* = 19)	C-PTSD cases (*N* = 3)
#1	13	0	0
#2	23	0	0
#3	12	0	0
#4	15	0	0
#5	9	0	0
#6	8	1	0
#7	8	1	0
#8	18	1	0
#9	13	1	0
#10	14	7	2
#11	8	8	1

## Discussion

As stated previously, the ACA and CBCL-DSM/ACA symptom profiles are differentiated more by symptom severity and complexity, than symptom specificity – suggesting that complex attachment- and trauma-related psychopathology is not amenable to traditional diagnostic classification. In other words, the profiles do not have sufficient symptom specificity to suggest a taxonomy of complex post-maltreatment developmental disorders. However, they offer an opportunity to test the validity of the BPD and C-PTSD constructs, against empirical, atheoretical symptom profiles. The ACA was designed to measure a broad range of maltreatment-related symptoms not adequately covered by general mental health measures such as the CBCL (Tarren-Sweeney, 2013a). The ACA symptom profiles were identified empirically through cluster analyses of the ACA scales, which in turn had been derived through factor analysis. The CBCL-DSM/ACA symptom profiles are less empirically ‘pure’, in that they were constructed from scores on five ACA scales and five CBCL DSM-oriented scales, with the latter being derived deductively rather than inductively.

The present findings do not support the validity of the C-PTSD construct for describing complex and severe psychopathology among adolescents with a history of maltreatment. Despite the concern that our method may have over-estimated PTSD caseness by defining cases from only two of the three diagnostic criteria, just 1.3% of this high-risk adolescent sample were nominal C-PTSD cases. What might explain this? One possibility is that our method under-estimated the number of participants meeting one or more diagnostic criteria, due to cut-points being set too high, and/or a lack of adequately matched items. This is critical given that C-PTSD diagnosis requires all of the diagnostic criteria to be met. However, the proportions of participants meeting the various diagnostic criteria ranged from 8 to 30% (see Table 3), suggesting there was no diagnostic ‘trip point’ causing under-detection of C-PTSD. For the PTSD criteria (#1 and #3), the relatively low 2 + cut-points yielded fairly high criterion assignment rates of 12% and 16% respectively, with 23% of the sample (*N* = 53) meeting one or both PTSD criteria. Surprisingly however, less than a quarter of those (23%, *N* = 12) met both PTSD criteria. We also believe that the selected ACA and CBCL items matched the C-PTSD criteria well. Instead, the very low nominal C-PTSD prevalence appears to be accounted for by low concordance across the five criteria, and more particularly, between PTSD and DSO caseness.

The present findings suggest that the core features of BPD (emotional and behavioural dysregulation, including impulsivity and intense anger; intense and unstable interpersonal relationships underpinned by fears of rejection and abandonment; disturbance of ‘self’ with negative self-image; dissociation and paranoia; and suicidality) are components of severe and complex developmental symptomatology experienced by adolescents who experienced early social adversity, including maltreatment. Of the 19 nominal BPD cases, 18 belonged to the three most severe and complex ACA clusters (ACA profiles #6, #7, #8). The most striking finding is that all 12 participants whose symptoms fitted ACA symptom profile #8 (the most severe and complex profile) were BPD cases, inferring that ACA profile #8 corresponds to severe or pronounced BPD. There was similar alignment of BPD caseness with the CBCL-DSM/ACA symptom profiles. Of the 19 BPD cases, 15 were assigned to CBCL-DSM/ACA symptom profiles #10 and #11 (the two most severe and complex profiles), and all eight participants that had the most severe profile (#11) were BPD cases, inferring that ACA-CBCL profile #11 corresponds to severe or pronounced BPD. The ACA symptom profiles #6 and #8 were characterised by co-occurring non-reciprocal social behaviour and intense and unstable interpersonal relationships underpinned by fears of rejection and abandonment (as measured by the SI-BD and ED-DSC scales). While these two sets of behaviours appear contradictory, they are consistent with a characteristic relational pattern observed among individuals with BPD, namely rapid alternation between craving contact and affection, and rejecting contact – and corresponding shifts between extremes of idealization and devaluation (Newman & Stevenson, 2005).

### Limitations and Implications for Future Research

The present study findings are based solely on caregiver-reported mental health checklist scores. Further research on this topic should ideally include adolescent clinical interviews and self-report measures, as well as caregiver-report measures. However, it is important to retain caregiver measures in any future study, given evidence that young people in OOHC systematically under-report their mental health difficulties in population studies (Tarren-Sweeney, 2019). A second possible study limitation is that BPD and C-PTSD cases were defined from items on broad-range mental health checklists, rather than instruments designed explicitly to measure BPD and C-PTSD diagnostic criteria. However, with the exception of C-PTSD criterion #2 (avoidance of thoughts and memories), we believe the items selected from the CBCL and ACA were, in the main, well matched to the various diagnostic criteria.

The present study focussed exclusively on the mental health outcomes of adolescents in foster care, without referencing other developmental outcomes, notably cognitive abilities and language. Neurocognitive deficits are likely to increase chronically maltreated children’s vulnerability for complex and severe mental health difficulties. Future investigations of complex symptomatology with maltreated children and adolescents would ideally include formal cognitive assessment, as well as neuropsychological, adaptive behaviour and language measures. Furthermore, longitudinal studies are needed to account for and disentangle complex transactional developmental processes affecting this population, including differentiating the effects of pre-care maltreatment from within-care adversity (e.g. placement instability, and relational impermanence), as well as the overlaying influence of emerging mental illness in adolescence.

### Practice Implications

Accurate assessment and formulation of complex symptomology among this vulnerable population is critical for treatment planning. The present study findings highlight the limitations of the ICD and DSM classification systems for young people with complex maltreatment-related symptomatology. While the terms ‘complex trauma’ and ‘developmental trauma’ are increasingly used to describe the clinical-developmental effects of early and prolonged maltreatment, the present study findings suggest a need for caution when diagnosing ‘trauma’ disorders among maltreated young people with complex difficulties. In particular, a thorough assessment of attachment-related relational difficulties and styles is necessary before diagnosing C-PTSD.

## Conclusion

Our investigation of alignment between empirically-derived symptom profiles and nominal diagnosis of DSM-V BPD and DSM-11 C-PTSD provides some support for the validity of the BPD construct among adolescents exposed to early maltreatment – but no support for the C-PTSD construct. While the complex and severe presentations identified in the present study included core symptoms and clinical signs of BPD, they were also characterised by clinical-level inattention/over-activity and conduct problems. The complex symptom profiles thus point to symptomatology that is broader and more complex than that encapsulated by the BPD construct. The present results therefore suggest that, while BPD *as it is presently conceptualised* describes a large portion of symptoms present in complex and severe adolescent presentations, it fails to account for co-occurring clinical-level conduct problems and inattention/over-activity (see Fig. 2). Given the high rate of co-morbid diagnosis of BPD with other developmental disorders (including ADHD) among adults, the present findings suggest that BPD may not be accurately conceptualised for adults who experienced childhood maltreatment. Otherwise, might the present findings point to the existence of an *adolescent complex disorder* that incorporates core features of Conduct Disorder, ADHD and BPD? While this is a tempting possibility, not least because it would provide clinicians a discrete diagnostic formulation that is unencumbered by co-morbidity, it is compromised by very high variability in combinations of multiple symptom types that does not conform to traditional definitions of a ‘diagnosable’ mental disorder. Furthermore, there may be greater utility in conceptualizing complex presentations among this population using a ‘symptom profile’ or ‘complex developmental formulation’ approach, in place of a traditional diagnostic formulation – that emphasises a trans-diagnostic focus on transactional developmental mechanisms of early caregiving, attachment, traumatic abuse, social learning conditions and biological vulnerability for emerging mental illness.
